# Role of Transluminal Balloon Angioplasty for the Treatment of Vasospasm Due to Aneurysmal Subarachnoid Haemorrhage: A Multicentric Indian Experience

**DOI:** 10.7759/cureus.29311

**Published:** 2022-09-19

**Authors:** Sibasankar Dalai, Uday S Limaye, Mohan V. Sumedha Maturu, Satya Rao Kolli, Rajesh Pati, Madhusudhana Babu Marthati, Sailesh Modi, Aravind Varma Datla, Sameera Anantamakula, Rajasekhar Donkada

**Affiliations:** 1 Interventional Neuroradiology, Medicover Hospitals, Visakhapatnam, IND; 2 Interventional Neuroradiology, Lilavati Hospital, Mumbai, IND; 3 Neurology, Medicover Hospitals, Visakhapatnam, IND; 4 Neurology, Queens NRI Hospital, Visakhapatnam, IND; 5 Internal Medicine, Medicover Hospitals, Visakhapatnam, IND; 6 Radiology, Medicover Hospitals, Visakhapatnam, IND; 7 Anaesthesiology, Medicover Hospitals, Visakhapatnam, IND

**Keywords:** dsa digital subtraction angiography, ruptured cerebral aneurysm, balloon angioplasty, endovascular angioplasty, endovascular procedures, cerebral vasospasm, delayed ischemic neurological deficit, aneurysmal subarachnoid haemorrhage

## Abstract

Background

Aneurysmal Subarachnoid Haemorrhage (aSAH) is a complex and critical neurological condition associated with significant mortality and morbidity. Apart from the initial insult due to the aneurysmal rupture itself, re-bleeding and severe cerebral vasospasm are some of the complications of aSAH that result in overall poor outcomes. Cerebral vasospasm in post-aSAH can result in delayed ischaemic neurological deficits. In the absence of timely interventions, it can lead to grave consequences for the patient. Management of cerebral vasospasm has been evolving over the years to prevent mortality and morbidity in aSAH patients.

Materials and methods

During 36 months from January 2018 to December 2020, 164 patients were admitted with aSAH in multiple Indian centres. Endovascular methods were used to treat all the aneurysms. Patients were observed for clinically symptomatic cerebral vasospasm. Patients with suspected vasospasm were further evaluated with a transcranial Doppler (TCD), brain computed tomography (CT) or magnetic resonance imaging (MRI) scan. In addition, digital subtraction angiography (DSA) of cerebral vessels was performed to evaluate vasospasm further. Twenty-two patients had clinically and angiographically significant vasospasm, and 20 patients were treated with transluminal balloon angioplasty (TBA).

Results

Satisfactory lumen dilation was achieved in 79 out of the 91 (86.81%) vasospastic segments, namely, distal internal carotid arteries (ICAs) 100%; middle cerebral arteries (MCA) 97.56% (M1=100%, M2=100%, M3=87.5%); vertebral arteries-100%; basilar arteries-100%; anterior cerebral arteries (ACA) 67.64% (A1=75%, A2=57.14%). The mean Modified Rankin Scale (mRS) score at 90 days was 0.75. 17 patients (85%) had an overall good outcome with no new neurological deficits. There were no cases of vessel rupture, dissection or thromboembolic complications.

Conclusion

TBA is a valuable, safe and effective option for managing clinically significant vasospasm caused by aSAH, adjuvant to medical management.

## Introduction

Aneurysmal Subarachnoid Haemorrhage (aSAH) occurs primarily due to ruptured saccular aneurysms, accounting for 5%-10% of all haemorrhagic strokes [1,2]. Globally, aSAH appears to have an incidence of five per 100,000 population [3]. It varies depending on the geographic area, and the highest rates are in North America and the lowest rates are in Asia outside Japan, and South and Central America [4]. Approximately 76,500 to 204,100 cases occur in a country like India annually [5]. The exact reasons for aneurysms are not known. However, female gender, smoking, cocaine abuse, a family history, and genetic conditions like Ehlers Danlos syndrome are some likely predisposing factors.

Cerebral vasospasm is a leading cause of delayed cerebral ischaemia, occurring around the 4-11th day of the ictus, and responsible for significant mortality and morbidity in such patients [6]. Vasospasm is believed to result from the direct effects of clotted blood and its breakdown products on the arteries within the subarachnoid space. In general, the more blood that surrounds the arteries, the greater the chance of symptomatic vasospasm. Spasm of major arteries produces symptoms referable to the appropriate vascular territory. All of these focal symptoms may present abruptly, fluctuate, or develop over a few days. In most cases, a focal spasm is preceded by a decline in mental status [7]. Such patients can also present with increased blood pressure, headache, and sensory and motor impairments.

For medically refractory cases, intra-arterial infusion of vasodilators and balloon angioplasty may be employed. The goal of endovascular treatment for symptomatic vasospasm is to increase cerebral blood flow and prevent infarction. Endovascular procedures such as the transluminal balloon angioplasty (TBA) approach are a valuable option after failure of medical management [8].

## Materials and methods

The present study retrospectively studied the procedural safety, and clinical efficacy of TBA in aSAH patients admitted for 36 months from January 2018 to December 2020 at various Indian centres. During this period, 164 patients (Lilavati Hospital Mumbai-47; Hinduja Hospital Mumbai-45; Seven Hills Hospital Visakhapatnam-53; Medicover Hospitals Visakhapatnam-19) were admitted with aSAH. Twenty-two patients developed clinically and angiographically significant vasospasm, and 20 (Mumbai centres-8; Visakhapatnam centres-12) were treated with TBA. In two of these patients, consent for the procedure could not be obtained. Two interventional neuroradiologists did all the procedures, each with 25 years and 15 years of experience, respectively.

Inclusion criteria

Patients classified under Fisher grading and those with grades III, and IV were included in the study only after securing the aneurysm.

Exclusion criteria

Patients who denied consent, patients with unsecured aneurysms, severe cerebral atherosclerosis, patients with medical conditions that could adversely affect mortality/morbidity, e.g., severe renal failure, poor Glasgow Coma Scale (GCS) scoring, etc.

For the 20 patients, inpatient and outpatient medical records, radiographic imaging, and procedural data analysis were done. Demographic features including age, gender, Fisher grade, aneurysm location, treatment approach, and timing of angioplasty were recorded. Clinical examination findings before and after the treatment were noted. TCD, CT, MRI, DSA were performed to determine cerebral vasospasm. The angiographic results were reviewed, and pre- and post-treatment vessel diameter, procedure-associated complications and clinical outcomes were assessed to determine efficacy and safety.

All patients were managed according to a standardised treatment protocol: aggressive resuscitation, including intubation, ventilation, hypertonic saline and mannitol administration as necessary. In all those admitted with aSAH, DSA was performed within 24 hours to detect the number, size, location, neck, and dome of the aneurysm to plan for further treatment. Endovascular therapy (ET) (coiling, balloon-assisted coiling, stent-assisted coiling, flow diversion, flow reversal or parent-artery occlusion) was done in all the patients to prevent re-bleeding. During the endovascular therapy (ET), intra-arterial nimodipine was administered via side flush for the guide catheter. All patients underwent post-procedure angiography to evaluate the result of the ET, unintentional vessel occlusion, or the presence of early vasospasm. All patients were monitored in intensive care units with close neurological monitoring and blood pressure monitoring for 11 days. They were all given volume expanders and oral nimodipine 60mg four-hourly from day 0. Patients who showed signs of neurological deterioration like a drop in sensorium (fall in GCS), hemiparesis, slurring of speech and vision abnormalities were immediately evaluated with a TCD, CT or MRI scan (to exclude hydrocephalus, bleeding or infarction). Those patients with a diagnosis of cerebral vasospasm (TCD blood velocities >200 cm/s in at least one intracranial vessel segment; or CT/MRI showing early ischemic changes) were subjected to a DSA. In patients who developed significant vasospasm compared to initial angiograms, angioplasty was performed at the earliest.

The vasospasm was considered significant if there was a decrease in the vessel diameter >50% compared to the native vessel (in DSA) associated with focal neurological deficits that correlate to reduced blood flow in the territories distal to the vasospasm. 

Criteria for diagnosing delayed ischemic neurological deficit (DIND) due to vasospasm were the onset of symptoms between days 3 and 14 after SAH, signs of worsening of headache, neck stiffness, confusion, disorientation, declined consciousness, focal neurological deficits referable to the vasospastic vessel. Fall in GCS score at least 2 points and radiological confirmation of vasospasm were also considered.

The outcomes were considered favourable if there is a clinical and angiographic improvement, no new neurological deficit, no recurrence of cerebral vasospasm in the following week, and mRS at 90 days ≤ 2.

Procedure

**Figure 1:**
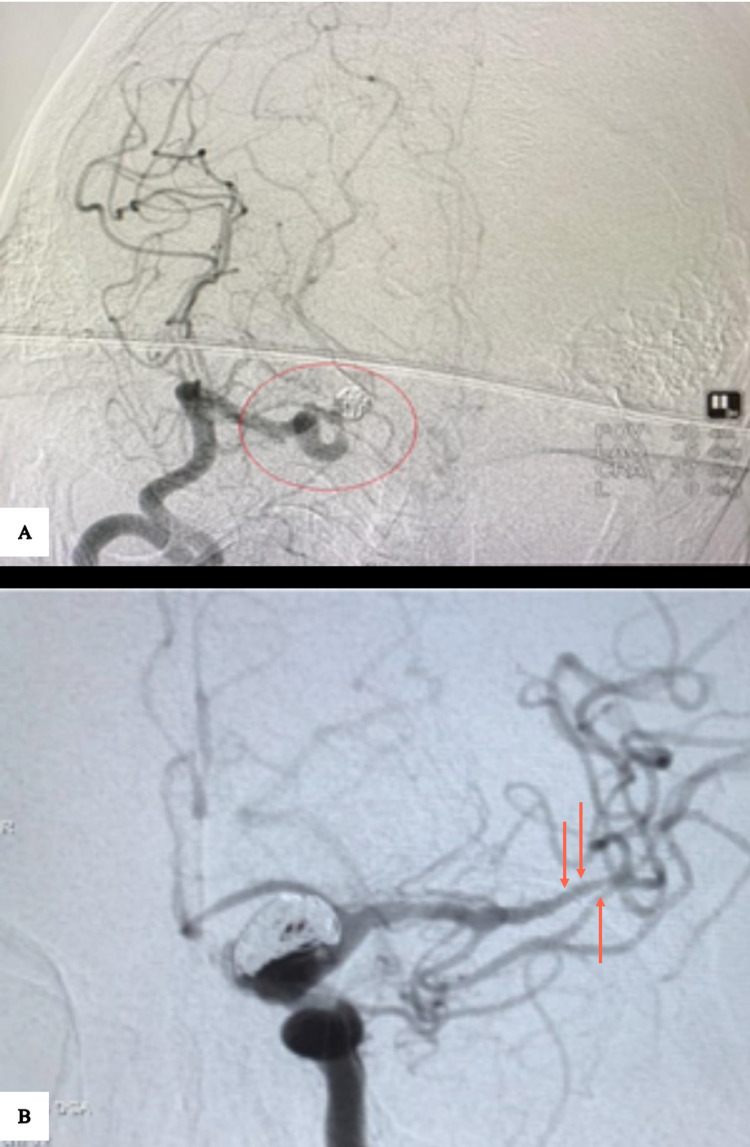
Cerebral DSA. (A) Vasospasm in the supraclinoid ICA and MCA (red outline) with a coiled aneurysm. (B) Vasospasm in the left MCA (red arrows) with a coiled aneurysm. DSA - Digital subtraction angiogram, ICA - Internal carotid artery, MCA - Middle cerebral artery

All angiography procedures were done by interventional neuroradiologists through the transfemoral arterial route, under general anaesthesia. All patients are given 3,000 units of heparin subcutaneously before the procedure. Activated Clotting Time (ACT) is monitored hourly, and the heparin dose is titrated to maintain an ACT between 160 and 200 seconds. A 7F long sheath is inserted into the affected vascular territory. A Distal Access Catheter, (DAC 6.4F - Stryker Neurovascular, USA), is positioned distally into the affected territory. An angiogram is performed to demonstrate the vasospasm (Figures 1A, 1B).

**Figure 2:**
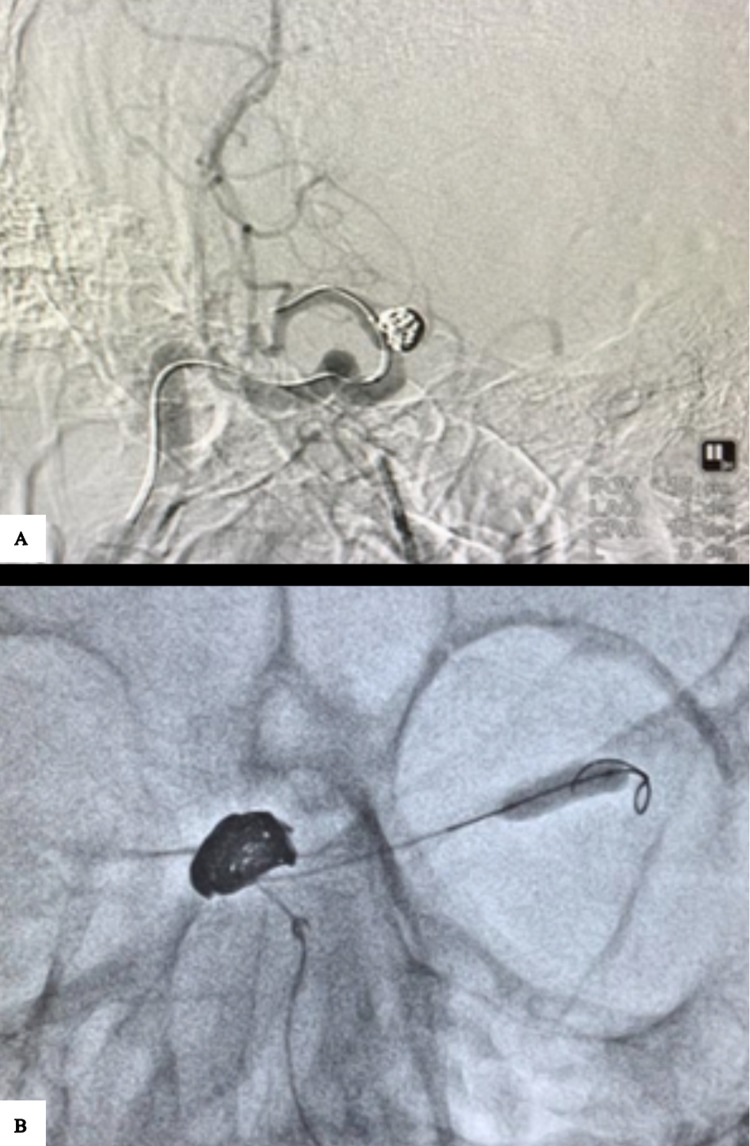
Cerebral DSA. Balloon wire navigated across the vasospastic segment (A). The balloon is dilated under roadmap guidance for 90-120 seconds (B). DSA - Digital subtraction angiogram

A balloon transform (Stryker Neurovascular, USA)/hyper glide (Medtronics Neurovascular, USA)/scepter C (Microvention Neurovascular, USA) is placed across the vascular segment affected by vasospasm (Figures 2A, 2B).

**Figure 3:**
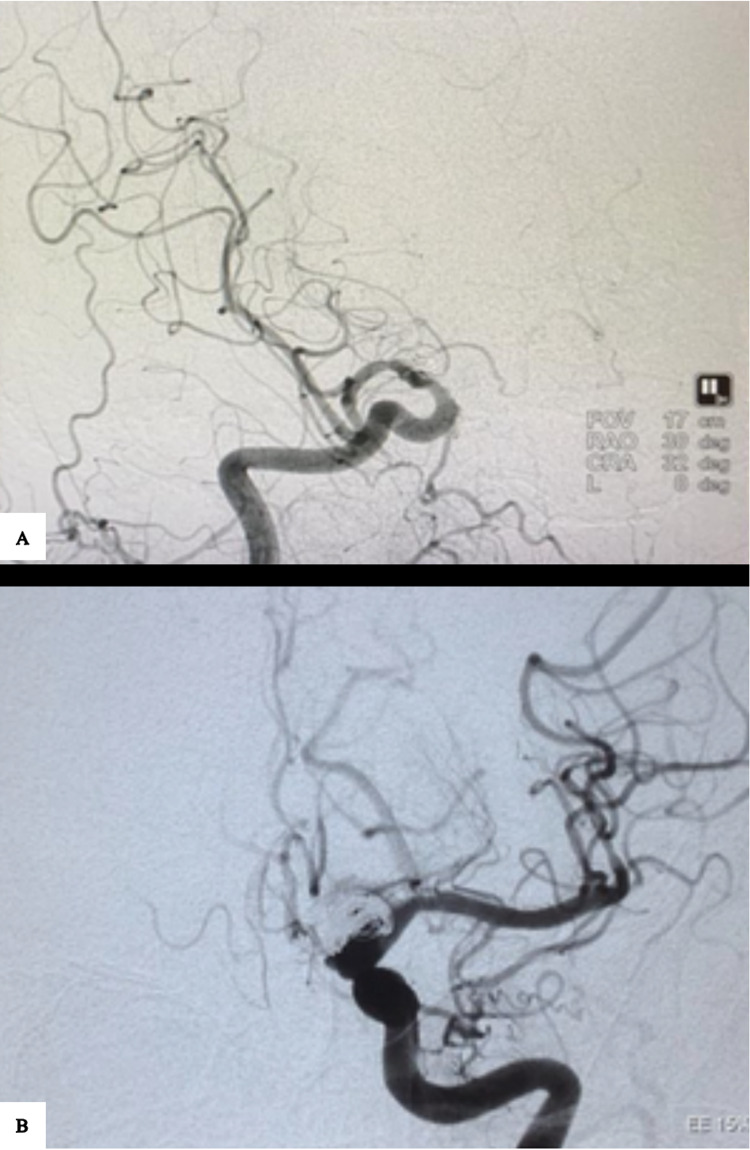
Cerebral DSA. Angiogram of the right ICA after angioplasty of the supraclinoid ICA and M1 segments of the MCA (A). Left MCA after angioplasty (B). DSA - Digital subtraction angiogram, ICA - Internal carotid artery, MCA - Middle cerebral artery

The size of the balloon is based on the native vessel diameter. Angioplasty is done, and the balloon inflation is maintained for approximately 90 to 120 seconds under roadmap guidance. After the balloon deflation, a check angiogram is done (Figures 3A, 3B).

The procedure is completed after satisfactory lumen dilation (approximately 80% of the native vessel diameter). The procedure is the same across all centres.

## Results

Out of the 20 participants in the present study (Table 1), eight were male (40%), and 12 were female (60%). The ages ranged from 36 to 72 years, with a mean age of 56.55 years. 55% of patients were of Fisher grade 4, whereas 45% had Fisher grade 3 subarachnoid haemorrhage (SAH). The locations of the aneurysms are the anterior communicating artery (40%), middle cerebral artery bifurcation (25%) and posterior communicating artery (35%). The mean time gap between SAH and TBA was 8.64 days. Satisfactory lumen dilation was achieved in 79 out of the 91 (86.81%) vasospastic segments. Distal ICA 100%; middle cerebral artery-97.56%; vertebral artery-100%; basilar arteries-100%; anterior cerebral artery-67.64% (Table 2).

**Table 1:** Demographic and radiological profile of the participants

Variable	Number (Frequency)
Age (years)	
35-50	3 (15%)
51-65	11 (55%)
>65	6 (30%)
Sex	
Male	8 (40%)
Female	12 (60%)
Fisher scale grading	
Grade 3	9 (45%)
Grade 4	11 (55%)
Location of aneurysms	
Anterior Communicating Artery	8 (40%)
Middle Cerebral Artery bifurcation	5 (25%)
Posterior Communicating Artery	7 (35%)
Overall outcomes	
Favourable	17 (85%)
Unfavourable	3 (15%)

 

**Table 2:** Distribution of vasospastic segments and treatment outcomes

Location of vasospasm	Number of vasospastic segments	Successfully treated vascular segments
Distal Internal Carotid Artery	13	13 (100%)
Middle Cerebral Artery		
M1	18	18 (100%)
M2	15	15 (100%)
M3	8	7 (87.5%)
Anterior Cerebral Artery		
A1	20	15 (75%)
A2	14	8 (57.14%)
Vertebral Artery	1	1 (100%)
Basilar Artery	2	2 (100%)

There was no recurrence of vasospasm in any of the participants. The mean pre-TBA GCS score was 13 ± 1.5. The mean post-TBA GCS score was 13.8 ± 1.91. The mean mRS at the time of discharge was 0.95. The mean mRS at 90 days was 0.75. In terms of overall outcomes, 17 patients showed clinical and radiographical improvements. In two patients, pre-TBA imaging revealed acute ischemic changes that could not be reversed despite immediate angioplasty. Of these two, one patient subsequently had a haemorrhagic transformation of the ischemic infarct. One patient on prolonged invasive ventilation developed ventilator-associated pneumonia, which evolved into septic shock leading to death.

## Discussion

Patients with aSAH experience vasospasm of large arteries. As a consequence of vasospasm, there is reduced distal perfusion pressure and autoregulatory dysfunction [9,10]. The vasospasm can be managed by inducing hypervolemic hypertension with intravenous volume expansion with crystalloids or colloids to increase cardiac output and raise blood pressure. However, small randomised trials showed no apparent benefit. Balloon and chemical angioplasty with a super-selective intra-arterial injection of vasodilators has emerged as the primary intervention for treating medically refractory ischemia from cerebral vasospasm. Some promising new treatments for cerebral vasospasm or its ischemic complications include magnesium sulphate, fasudil hydrochloride, tirilazad mesylate, erythropoietin, and induced hypothermia; however, they all still need further evaluation in clinical trials. Newly recognised mediators of cerebral vasospasm after SAH include endothelium-derived mediators, vascular smooth-muscle-derived mediators, cytokines and adhesion molecules, pro-inflammatory mediators involved in blood-brain barrier disruption, stress-induced gene activation, and platelet-derived growth factors. More extensive, prospective, randomised trials are needed to verify several hypotheses of molecular pathophysiology and clinical treatment regimens [11].

Though chemical angioplasty with intra-arterial nimodipine is a justifiable approach, the effects of such intervention are not persistent. In the study by Cho et al., 21 (50%) out of 42 patients underwent intra-arterial drug infusion more than once, with a mean session number of 2.4±1.9 [12].

In 1984, Zubkov and colleagues first reported treating aSAH patients using balloon catheters to dilate vasospastic arteries and demonstrated the technical capacity to treat stenotic vessels using endovascular procedures [13]. The typical structure of collagen fibres in the vessel walls was affected significantly by balloon dilatation. Moreover, the long-lasting effects of balloon dilatation may be caused by the disruption of connective tissues that proliferate in the vessel wall after a subarachnoid haemorrhage [14,15].

Three types of balloons are used in angioplasty. Non-compliant balloons are used for coronary and peripheral angioplasties. Within the cerebral vasculature, super-compliant balloons are utilised at vessel bifurcations, whereas compliant balloons are preferred in straight vessels.

Beck et al. showed that successful TBA improved tissue perfusion and prevented cerebral infarction [16]. In the studies by Eskridge et al. and Zubkov et al., vessel segments treated with angioplasty rarely developed re-stenosis to the point of warranting further therapy [13,17]. Multiple studies have demonstrated that angioplasty achieves long-lasting dilatation of spastic cerebral arteries [18-21]. However, Sedat et al. reported a case of asymptomatic and permanent re-stenosis after angioplasty for cerebral vasospasm [22]. Zwienenberg-Lee et al. described the superiority of TBA compared to pharmacological treatment, and its relatively low risk [21].

In our study, we dilated the lumen to approximately 80% of the native vessel diameter. This was done to decrease the chances of vessel rupture or dissection. Peri-procedural complications for balloon angioplasty can include vessel perforation, vessel dissection, thromboembolic events, device malfunctions, retroperitoneal hematomas, or groin hematomas [11]. We experienced no such events in our study.

In the reports by Terry et al., 75 patients underwent 85 TBAs for the treatment of SAH-induced vasospasm [20]. No vessel rupture or perforation occurred, and TBA was successful in the distal ICAs (100%), proximal middle cerebral (94%), vertebral (73%), and basilar arteries (88%) which are comparable to our study. However, TBA was successful in only 34% of anterior cerebral arteries, compared to 67.64% in our study. The severity of the vasospasm or an unfavourable angle was attributed as the cause. Similar observations were made in a retrospective study by Choi et al. with better success rates in the anterior cerebral artery territories [23].

Our study demonstrates the balloon angioplasty's safety and technical efficacy in most of the vasospastic segments, with a success rate of 100% for the distal internal carotid and vertebrobasilar arteries; 97% for middle cerebral arteries and 67% for the anterior cerebral arteries. In some patients, we could not navigate a balloon microwire into the A1 segment because of an unfavourable angle of the anterior cerebral artery origin. As observed by Terry et al. and Choi et al., the success rate for anterior cerebral arteries was lower than that found for other intracranial arteries, and this was found to be the result of an angle that prevented navigation of the balloon microwire [20,23]. Accordingly, the untreated A1 and A2 segments were treated by intraarterial nimodipine injection.

Hoh and Ogilvy estimated that 62% of patients improved clinically after TBA [24]. However, in the present study, a favourable outcome was observed in 85% of the participants. The advances in catheter technology, the experience of the operators, improvement in peri-procedural management, and prompt initiation of angioplasty could be the reason for the improved outcomes. The limitations of the study are its retrospective nature, non-randomization and the small number of participants. Large-scale randomised trials are needed to further verify the observations in our study.

## Conclusions

Cerebral vasospasm can result in delayed ischaemic neurological deficits, that significantly contribute to patient morbidity and mortality. A high level of vigilance is necessary to detect the early signs of focal neurological deficits and to initiate timely treatment. Intensive postoperative care and regular follow-up are vital for achieving the best possible outcomes. This study concludes that it is safe and efficacious to do balloon angioplasty in patients with clinically significant vasospasm induced by aneurysmal subarachnoid haemorrhage.
